# Efficacy of chordal cutting in alleviating ischemic mitral regurgitation: insights from 3-dimensional echocardiography

**DOI:** 10.1186/1749-8090-2-39

**Published:** 2007-09-25

**Authors:** Chittoor B Sai-Sudhakar, Rashmi Vandse, Todd A Armen, Katherine M Bickle, Nadia S Nathan

**Affiliations:** 1 Department of Cardiothoracic Surgery, Ohio State University Medical Center, N-816 Doan Hall, 410 W 10^th ^Avenue, Columbus, OH 43210, USA; 2 Department of Anesthesiology, Ohio State University Medical Center, N-416 Doan Hall,410 W 10^th ^Avenue, Columbus, OH 43210, USA

## Abstract

**Background:**

Ischemic mitral regurgitation often complicates severe ischemic heart disease and adversely affects the prognosis in these patients. There is wide variation in the clinical spectrum of ischemic mitral regurgitation due to varying location and chronicity of ischemia and anomalies in annular and ventricular remodeling. As a result, there is lack of consensus in treating these patients. Treatment has to be individualized for each patient. Most of the available surgical options do not consistently correct this condition in all the patients. Chordal cutting is one of the newer surgical approaches in which cutting a limited number of critically positioned basal chordae have found success by relieving the leaflet tethering and thereby improving the coaptation of leaflets. Three-dimensional echocardiography is a potentially valuable tool in identifying the specific pattern of tethering and thus the suitability of this procedure in a given clinical scenario.

**Case Presentation:**

A 66-year-old man with cardiomyopathy and ischemic mitral regurgitation presented to us with the features of congestive heart failure. The three-dimensional echocardiography revealed severe mitral regurgitation associated with the tethering of the lateral (P1) and medial (P3) scallops of the posterior leaflet of the mitral valve due to secondary chordal attachments. The ejection fraction was only 15% with severe global systolic and diastolic dysfunction. Mitral regurgitation was successfully corrected with mitral annuloplasty and resection of the secondary chordae tethering the medial and lateral scallops of the posterior leaflet of the mitral valve.

**Conclusion:**

Cutting the second order chordae along with mitral annuloplasty could be a novel method to remedy Ischemic mitral regurgitation by relieving the tethering of the valve leaflets. The preoperative three-dimensional echocardiography should be considered in all patients with Ischemic mitral regurgitation to assess the complex three-dimensional interactions between the mitral valve apparatus and the left ventricle. This aids in timely surgical planning.

## Background

Ischemic Mitral regurgitation (IMR) continues to be a complex surgical problem. Choosing the most optimal therapy remains a challenging issue in cardiac surgery. There is wide variation in the clinical spectrum of IMR due to varying location and chronicity of ischemia and anomalies in annular and ventricular remodeling. As a result, there is lack of consensus in treating these patients. Treatment has to be individualized for each patient. Three-dimensional (3-D) echocardiography is a potentially valuable tool in gaining the mechanistic insights into the pathogenesis of IMR by precise evaluation of the complex three-dimensional interactions between the mitral valve apparatus and the left ventricle. Noting the exact geometric perturbations in each individual clinical setting will enable us in the rational design of the specific repair methods and in making the choice amongst the different techniques and devices that are currently available. We present a case of severe IMR in which ring annuloplasty combined with chordal cutting was planned based on the findings on 3-D echocardiography with a good result.

## Case Presentation

**Figure 1:**
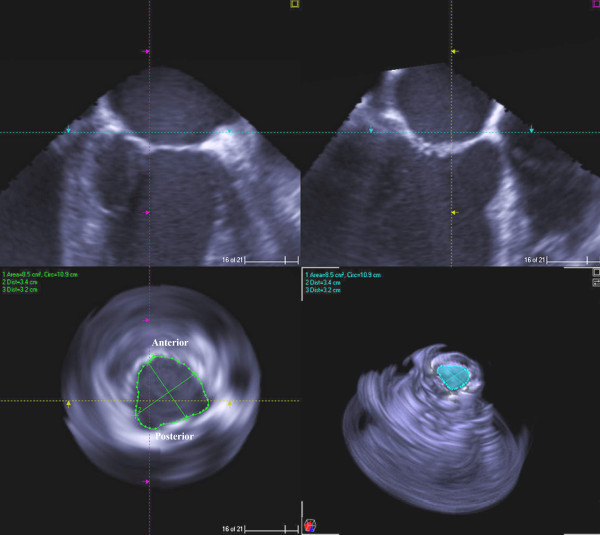
2D and 3D measurements of mitral valve circumference and area prior to the procedure.

**Figure 2:**
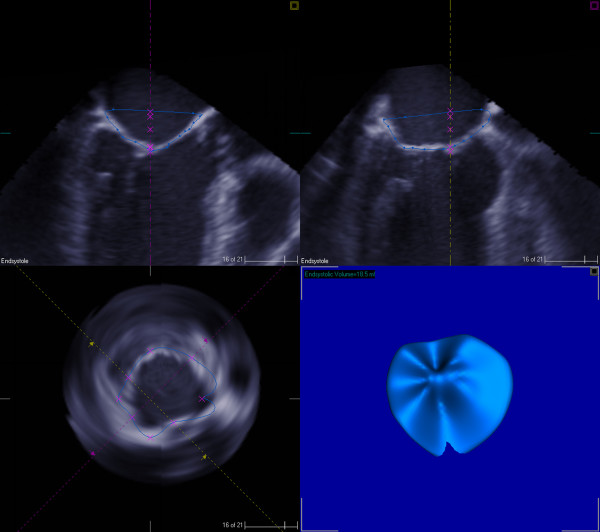
Preoperative 3D measurements of mitral annular volume (Measured from annulus to cusp).

**Figure 3:**
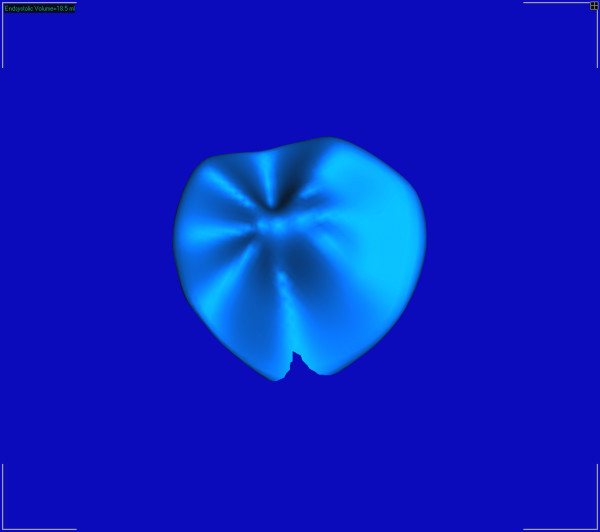
3D close up of mitral valve volume reconstruction showing tethering of the P1 and P3 leaflets.

A 66-year-old African-American male with cardiomyopathy and IMR, presented to the emergency room with a 3 day history of breathlessness and lower extremity edema. Chest radiography displayed marked cardiomegaly. A 2-dimensional echocardiography demonstrated severe (3–4+) mitral regurgitation, moderately severe aortic valve insufficiency, tricuspid regurgitation (3+) with moderate pulmonary hypertension (right ventricular systolic pressure was 58 mm of Hg). The LV was markedly dilated with the end diastolic diameter of 6.7 cm and end systolic diameter of 6.0 cm. The ejection fraction was only 15% with severe global systolic and diastolic dysfunction of the LV. Cardiac catheterization showed triple vessel disease with severe diffuse stenosis of the right coronary artery, left coronary artery and first diagonal artery. Intra-operatively, transesophageal 3-D echocardiography (TEE) was performed by multiplane TEE probe using Sonos^® ^7500 (Philips Medical Systems, N.A, Bothel, WA, USA). The 3-D processing was performed with 4-D Cardio-view^®^1.3 (TomTec Imaging Systems, Munich, Germany). The three-dimensional left ventricular model was generated. It produced a shell reconstruction of the LV allowing to calculate total and segmental left ventricular volumes during the cardiac cycle. The end systole frame was determined as frame with a closed aortic valve just prior to the opening of the mitral valve. This time point was used to make all valve measurements. Frame by frame analysis yielded the valvular area, circumference, commissure-to-commissure (c-c) diameter, antero-posterior (a-p) diameter (Figure 1) and mitral annular volume (measured from annulus to cusp) (Figure 2). The 3-D reconstruction of the mitral annular volume showed tethering of the lateral scallop (P1) and medial scallop (P3) of the posterior leaflet of the mitral valve due to the secondary chordal attachments. There was an eccentric jet due to inadequate apposition of the mitral leaflets. (Figure 3).

**Figure 4:**
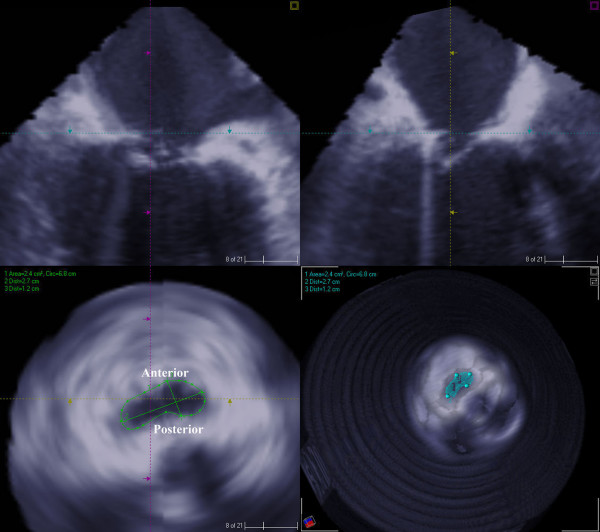
2D and 3D measurements of mitral valve circumference and area after chordal cutting and ring placement.

**Figure 5:**
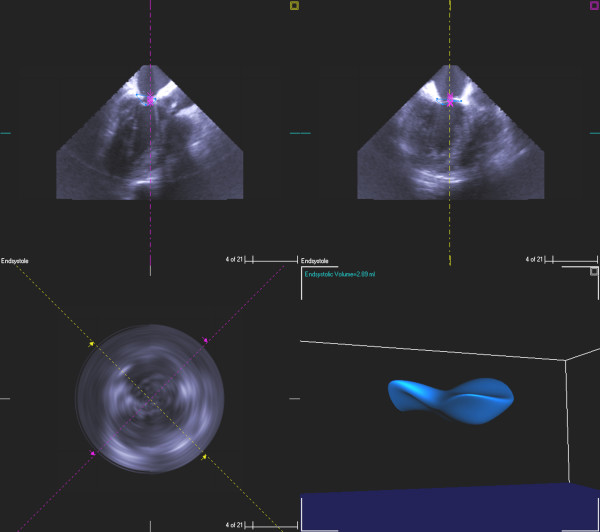
3D measurement of annular volume of mitral valve (measured from annulus to cusp) after chordal cutting and ring placement.

The surgical plan was to increase the zone of coaptation by chordal cutting to relieve the P1 and P3 tethering by the secondary chordae and performing a mitral annuloplasty. Coronary revascularization was performed along with the aortic valve replacement with a bioprosthetic valve, resection of the secondary chordae tethering the P1 and P3 scallops, mitral annuloplasty with a saddle ring and closure of a patent foramen ovale. In addition to intra-operative visualization and testing, the site of chordal resection was determined by the 3-D echocardiographic analysis of the tethered portions of the posterior leaflet of the mitral valve. At the conclusion of the operation and in the follow up period echocardiography demonstrated a competent mitral valve. There was a significant decrease in the mitral valvular area, circumference, commissure-to-commissure (c-c) diameter, antero-posterior (a-p) diameter (Figure 4) and mitral annular volume (Figure 5) post operatively.

The numerical data comparing the pre- and post-operative measurements of mitral valve is presented in Table 1.

**Table 1:** Showing the pre- and post-operative measurements of Mitral valve

	Valve Area (cm^2^)	Circumference (cm)	Annular Volume (mL)
Pre-operative	8.5	10.9	18.5
Post-operative	2.4	6.8	2.9

## Discussion

Ischemic mitral regurgitation (IMR) often complicates severe coronary artery disease and has a negative impact on the long term survival of these patients. Several anatomical and pathophysiological changes play a role in the genesis of IMR. The initiating event is an ischemic insult to the three dimensional interaction between the leaflets, annulus, subvalvular apparatus and left ventricular wall. The remodeling of the ischemic left ventricle results in increase in the sphericity index [1,2], mitral annular dilatation [3,4], and outward displacement of the papillary muscles with the subsequent tethering and tenting of the mitral leaflets into the left ventricle [5-8]. All these have been implicated in the pathogenesis of IMR. In the setting of left ventricular systolic dysfunction, there is also reduction in the force available to close the leaflets in opposition to the increased tethering which further complicates the IMR [9-11]. Mitral regurgitation in turn leads to a vicious cycle of volume overload, with further progression of annular dilation, LV remodeling and increased severity of symptoms of congestive heart failure. Mitral regurgitation is an independent predictor of mortality and adversely affects the survival and functional status [12-15].

Normal mitral valve has a unique morphology with a non-planner saddle shape and curved mitral leaflets. [16-18]. The 3-dimensional configuration of the curved mitral annulus is difficult to investigate using conventional 2-D echocardiography as the images represent a single plane which requires mental reconstruction of the 2-dimensional views [16,17,19]. Characterization of the mitral valve apparatus using 3-D echocardiography has shed a new light on the pathophysiology of mitral regurgitation [5-9], [20-23]. One of the important predictors of IMR is tenting of the mitral valve [5-9,24]. Tenting results from tethering of the leaflets, which is an end result of the perturbation in the complex three-dimensional interactions between the mitral valve apparatus and the left ventricle. The accuracy and variability of conventional 2-D echocardiography in these patients is often limited as it requires geometrical assumptions of 2-dimensional views. As a result some spatial relations and different structural features can be perceived erroneously even by the experienced echocardiographers. This would call for an unexpected modification in the technique of repair in the operating room, where the surgeon is challenged by limited time, operating field and non-physiological condition of the heart being devoid of blood which does not mimic the normal hemodynamic situation. In this clinical setting, the 3-D echocardiography is a potentially valuable tool as it creates images, resembling the true anatomy of the heart. By choosing a cutting plane and reconstructing the image beyond this plane the heart can be opened as if by a surgeon. This enhances the ability to assess the spatial relations between cardiac structures and also the specific tethering patterns.

Remodeling anunuloplasty using an undersized ring is the standard therapeutic approach for MR. It reduces antero-posterior valve dimensions by bringing the posterior annulus forward thus increases coaptating surface of the leaflets [1,25-27]. However, several studies have demonstrated that MR can persist or recur even after ring annuloplasty. This could be due to the fact that annuloplasty doesn't sufficiently address the tethering of the leaflets by the remodeled ventricle [28-31]. Chordal cutting, papillary muscle imbrication in combination with LV volume reduction, Septal anterior ventricular exclusion (SAVE), papillary muscle sling, ventricular reconstruction, pericardial patch enlargement of the restricted PML are some of the strategies that have shown success in limited number of patients [32,33]. Chordal cutting approaches championed by Messas et al [34] have shown promising results in several experimental and clinical studies [32,34-36]. In these studies, cutting a limited number of critically positioned basal chordae has shown to improve the coaptation by relieving the leaflet tethering and thereby reduce chronic persistent IMR even in the remodeled ventricle. Subsequently, this therapeutic option was challenged by another experimental study by Rodriguez et al [37] in which the chordal cutting did not relieve IMR. This controversy could be better understood by examining the specific valvular geometry through 3-D echocardiography.

### Technical limitations of 3-D echocardiography

The 3-D images were actually reconstructed from the multiplanar 2-D images taken over several cardiac cycles. Therefore, 3-D TEE reconstructions depend critically on the quality of the original 2D sectional images. There is a risk of introducing artifacts from patient respiration, motion and irregular movements of the heart during arrhythmias. Analysis was performed off-line and required tedious manual tracing of endocardial borders. The time required for reconstruction is an important factor limiting its applicability in the operating room [38]. However, the past years have seen significant development in transducer technology, allowing pyramidal shaped blocks to be acquired in real time. It is now possible to image an entire ventricle or valve and analyze it online, thus obviating the need for complicated and time-consuming reconstructions. However, Images obtained by Real-time 3-D echocardiography are of poorer quality compared to conventional 2-D echocardiography [21-23,39].

## Conclusion

All patients with IMR should be considered for pre-operative 3D TEE evaluation for timely surgical planning and also for intra-operative 3D TEE imaging and reconstruction to assess the dynamic changes that occur intra-operatively.

## Competing interests

The author(s) declare that they have no competing interests.

## Authors' contributions

RV contributed to the conception and design of the manuscript, review of literature and drafted the manuscript, TA, NN gave the anesthesia for the case, TA, NN and SS did the echocardiography of the case and its analysis and obtained the 3-D images and revised the manuscript for its important intellectual content, NN gave the final approval of the version to be published, KB participated in the data acquisition and writing the paper along with the first author. All authors read and approved the final manuscript.
